# In vivo tracking of tau pathology using positron emission tomography (PET) molecular imaging in small animals

**DOI:** 10.1186/2047-9158-3-6

**Published:** 2014-03-15

**Authors:** Eduardo Rigon Zimmer, Antoine Leuzy, Venkat Bhat, Serge Gauthier, Pedro Rosa-Neto

**Affiliations:** 1 Translational Neuroimaging Laboratory (TNL), McGill Center for Studies in Aging, Douglas Mental Health University Institute, McGill University, 6875 La Salle Blv - FBC room 3149, Montreal, QC, H4H 1R3, Canada; 2 Montreal Neurological Institute (MNI), Montreal, Canada; 3 Department of Biochemistry, Federal University of Rio Grande do Sul (UFRGS), Porto Alegre, Brazil; 4 Department of Psychiatry, McGill University, Montreal, Brazil

**Keywords:** Positron emission tomography, Tau molecular agents, Tau rodent models, Tauopathies

## Abstract

Hyperphosphorylation of the tau protein leading to the formation of neurofibrillary tangles (NFTs) is a common feature in a wide range of neurodegenerative diseases known as tauopathies, which include Alzheimer’s disease (AD) and the frontotemporal dementias (FTDs). Although heavily investigated, the mechanisms underlying the pathogenesis and progression of tauopathies have yet to be fully understood. In this context, several rodent models have been developed that successfully recapitulate the behavioral and neurochemical features of tau pathology, aiming to achieve a better understanding of the link between tau and neurodegeneration. To date, behavioral and biochemical parameters assessed using these models have been conducted using a combination of memory tasks and invasive methods such as cerebrospinal fluid (CSF) sampling or post-mortem analysis. Recently, several novel positron emission tomography (PET) radiopharmaceuticals targeting tau tangles have been developed, allowing for non-invasive *in vivo* quantification of tau pathology. Combined with tau transgenic models and microPET, these tracers hold the promise of advancing the development of theoretical models and advancing our understanding of the natural history of AD and non-AD tauopathies. In this review, we briefly describe some of the most important insights for understanding the biological basis of tau pathology, and shed light on the opportunity for improved modeling of tau pathology using a combination of tau-radiopharmaceuticals and animal models.

## Introduction

Tau is a microtubule-associated protein (MAP) responsible for the maintenance and promotion of cell microtubule stability. In the human brain there are six tau isoforms generated by in or out exons splicing and expressed as three repeat (3R) and four repeat (4R) isoforms. In the normal human brain these isoforms are present at similar levels [1]. Several serine (Ser) and threonine (Thr) phosphate acceptor residues (almost 90 sites in the longest form of human tau) are the main regulators of tau functioning and are a target for a wide range of brain kinases involved in crucial signaling pathways owing to the high availability of these phosphorylation sites [2].

There are at least four key kinases regulating tau phosphorylation states: glycogen synthase kinase-3B (GSK3-B), cyclin-dependent kinase 5 (CDK5), cAMP-dependent protein kinase (PKA) and microtubule-associated regulatory kinase (MARK). In addition to mediating axonal cytoskeletal stability, tau is involved in axonal development—including axonogenesis, polarization, outgrowth and myelination [3]—as well as in neuronal plasticity and the integration of microtubule functions with interneuronal signaling pathways [3,4].

**Figure 1:**
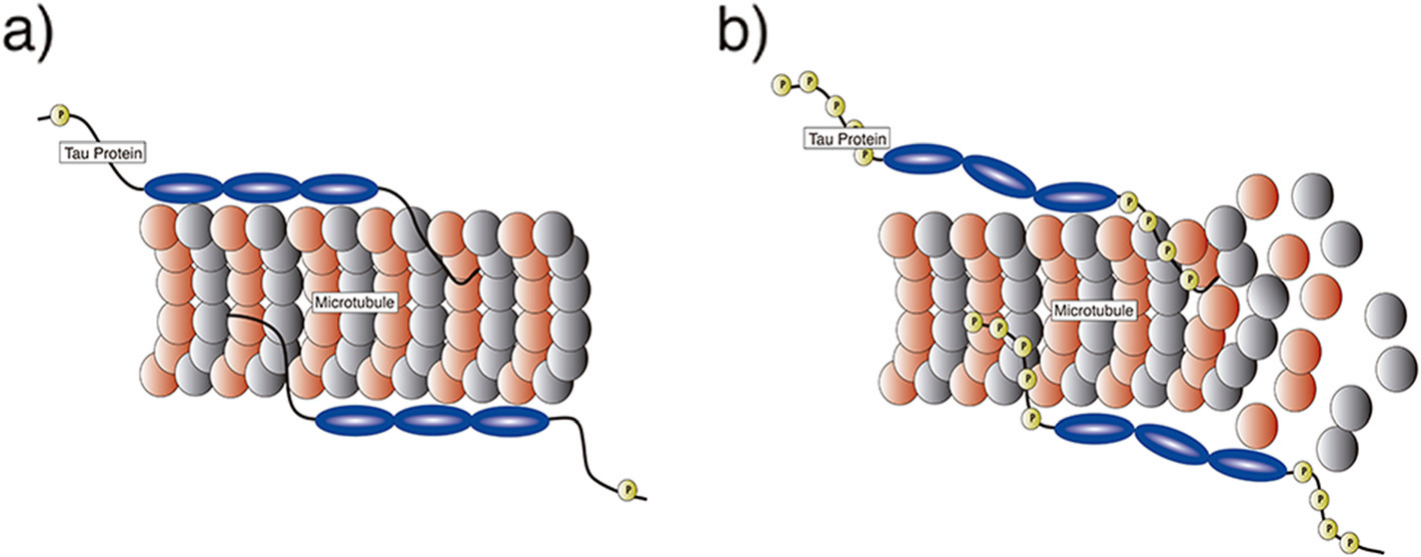
**Tau protein physiological functions and pathological role. a)** Physiological function: Tau protein promotes assembly and stabilization of microtubule architecture. Tau phosphorylation sites and isoforms are in charge of regulating tau function; **b)** Pathological role: Aberrant phosphorylation at various serine/threonine sites attenuates tau binding, which results in microtubule destabilization followed by accumulation in paired helical filaments (PHFs) and formation of insoluble aggregates (neurofibrillary tangles; NFTs).

The fine-tuning of tau phosphorylation also depends on protein phosphatase activities, in particular protein phosphatase 2A (PP2A). The major phosphatase in the human brain [5], PP2A accounts for the overwhelming majority of tau dephosphorylation. In fact, tau homeostasis depends on kinase/phosphatase activities and is of vital importance for the maintenance of microtubule stability, dynamics, as well as neuronal viability (Figure 1a).

### Seeding and spreading of tau pathology

Aberrant phosphorylation of tau leads to disruption of microtubule stability and neurodegeneration. Phosphorylation in the residues Ser199/Ser202/Thr205 (AT8), Thr212/Ser214/Thr217 (AT100), Thr231/235 (AT180) and Ser396/Ser404 (PHF-1) are invariably abnormal in pathological processes involving tau [6] (Figure 1b). Abnormally phosphorylated tau undergoes misfolding and aggregation, which constitutes a widespread feature among neurodegenerative diseases, including Alzheimer’s disease (AD) and Frontotemporal dementias (FTDs) [7,8].

**Figure 2:**
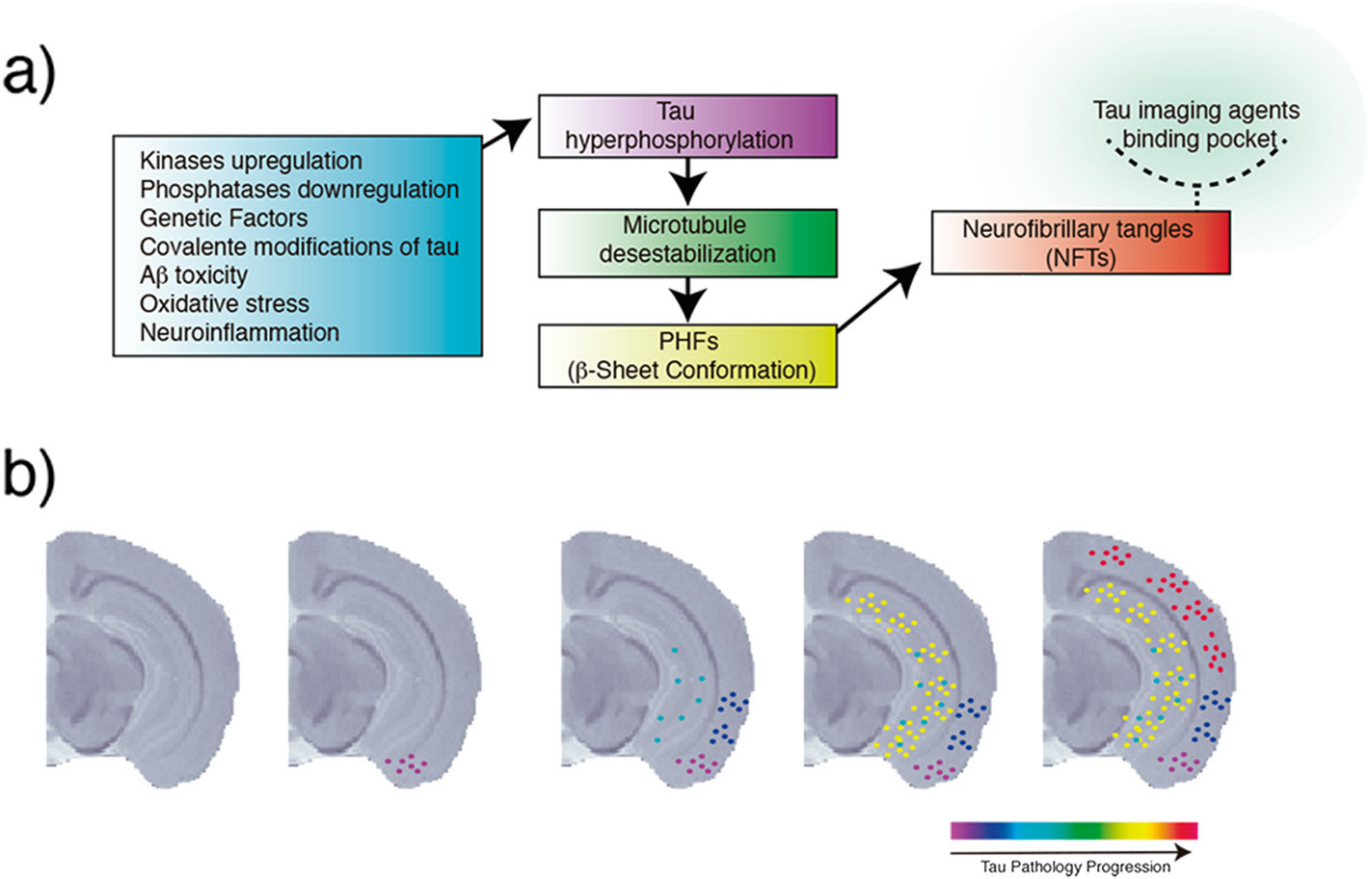
**Neuropathological mechanisms possibly involved in tauopathies and expected tracking of tau spreading by imaging agents in rodent models. a)** Pathological events (blue box) that contribute to tau hypherphosphorylation (purple box), microtubule detachment (green box), aggregation of paired helical filaments (PHFs) containing β-sheet structures (yellow box) and formation of neurofibrillary tangles (NFTs) containing the binding pocket for tau tracers (red box); **b)** Expected progression of tau pathology as shown by tau imaging agents based on previous findings in AD-like mouse models using immunohistochemistry. The image represents a coronal view of a T1-weighted mouse brain structural magnetic resonance imaging (MRI) image at bregma -2.88 mm.

In these diseases, the so-called tauopathies, tau deposits are commonly found in the cell bodies and neuronal dendrites, assuming a fibrillar conformation known as neurofibrillary tangles (NFTs) [9] (Figure 2a). In AD, NFT formation follows a well-characterized pattern of propagation, first described by Braak and Braak. The Braak staging follows the topographical evolution of the disease by means of six stages, which demonstrate the spread of tau pathology from the transentorhinal layer (I-II) toward limbic (III-IV) and isocortical areas (V-VI) [10]. Additionally, several studies have shown the degree of tau pathology to correlate with synaptic dysfunction and brain atrophy, as well as the severity of cognitive decline [11-13]. Importantly, while in AD tangles are constituted of equimolar 3R and 4R isoforms, FTD variants express predominantly 3R or 4R isoforms [14].

The main challenge with respect to defining the role of tau pathology in human neurodegenerative conditions is the co-existence of multiple pathology types. For example, in AD it is hard to develop theoretical models that allow for predictions regarding the evolution of tau pathology since amyloidosis is also present to an important degree. To overcome the effect of multiple pathologies, animal models expressing a single human tau mutation or displaying aberrant tau phosphorylation can be used as a method of choice to achieve an improved understanding of tau biology, pathology and the mechanisms underlying its spread throughout the brain.

### Genetic and chemical models of tau pathology

The use of rodents for recapitulating human neurodegenerative diseases has been unquestionable in providing new insights at a mechanistic level, with various genetic and chemical models capable of mimicking different features of tau pathology [15]. More specifically, the majority of transgenic models are constructed based on tau pathogenic mutations from frontotemporal dementia with parkinsonism-17 (FTDP-17), producing 4R pathology [16-18]. In addition, transgenic models using non-mutated human truncated tau are able to recapitulate neurofibrillary degeneration [19]. Recent advances, aiming to better model neurodegenerative changes seen in AD, pieced together mutated human amyloid precursor protein (hAPP) and wild-type human tau (without pathogenic mutation), producing a model displaying amyloid plaques and NFTs containing both 3R and 4R isoforms [20].

Importantly, transgenic models expressing human tau are capable of reproducing NFT formation [21,22]. In contrast, genetic models lacking human tau expression such as p25-transgenic mice, which overexpress the human activator of CDK5 kinase, [23,24] and chemical-induced models [25,26] lead to hyperphosphorylated tau in brain regions rich in tau expression, but not to NFT formation (for review of tau rodent models see http://www.alzforum.org/research-models).

Tau models have been employed for analyzing mechanisms, pathophysiology, behavior, and also for testing novel therapeutic strategies. Currently, behavioral tasks, analyzed by several memory paradigms, such as the Morris water maze (spatial memory), object recognition task (recognition memory) and Y or T-maze (working memory), have been correlated with cerebrospinal fluid levels of tau and phospho-tau. Similar to humans, animal cognition correlates with tau pathology quantified in post-mortem tissue [25,27-29]. However, in terms of assessing the role of tau burden on cognition, classic behavior-pathology correlation methods are of limited use due their invasiveness and intrinsic cross-sectional study designs. In contrast, Positron Emission Tomography (PET) using tau-imaging agents provides a unique opportunity to observe the progression of tau pathology non-invasively and longitudinally.

### Tau imaging agents

Advances in radiopharmaceuticals have recently led to novel tau-imaging agents, including [^18^ F]T807, [^18^ F]T808, [^18^ F]THK523, [^18^ F]THK5105, [^18^F]THK5115 and [^11^C]PBB3. In the following paragraphs, we briefly discuss the state-of-the-art of these compounds and what lies ahead.

[^18^ F]T807 and [^18^ F]T808 probes show promising results for tracking tau pathology in early clinical studies. In addition, post-mortem autoradiography followed by immunohistochemistry shows co-localization between these radiopharmaceuticals and phospo-tau antibodies [30,31].

Similar autoradiography studies with fluorine-tagged ligands THK523 (first-generation), THK5105 and THK5117 (second-generation) show high affinity for tau fibrils [32]. [^18^ F]THK523 shows specificity for tangles in studies conducted in transgenic mice models harboring only amyloid plaques or NFTs [33]. However, similarly to several [^18^ F] tracers, [^18^ F]THK523 shows an undesired high non-specific retention in white matter (WM) that may obscure cortical uptake. WM non-specific uptake reduces the diagnostic sensitivity of numerous imaging agents. To overcome this limitation, the second-generation of THK tracers, [^18^ F]THK5105 and [^18^ F]THK5117, were optimized for higher NFT cortical uptake and less WM retention [34].

The only tau radioligand labeled with carbon-11, [^11^C]PBB3, shows striking specificity for NFT detection in transgenic models overexpressing human tau mutation. Despite its structural similarities with [^11^C]PiB, the [^11^C]PBB3 data obtained in transgenic mice support specificity for tau pathology. In humans, [^11^C]PBB3 shows cortical uptake consistent with Braak staging. Indeed, positive [^11^C]PBB3 and negative [^11^C]PiB characterized a corticobasal syndrome patient [35]. These tracers hold the promise of allowing for the *in vivo* detection and tracking of tau pathology, and may prove of use in differentiating AD and non-AD tauopathies. However, it is not entirely clear whether a single tau-imaging agent will be useful to quantify pathology underlying the entire spectrum of tauopathies given the presence of molecular heterogeneity. For example, it is commonly known in PET literature that the interaction between an imaging agent and a molecular target (e.g. a protein) occurs only if the binding pocket is accessible to the imaging agent. Binding pocket availability is highly dependent on the protein structural conformation, which can be absent if the protein assumes an unusual conformation. Consequently, the ability of tracers to bind to NFTs may be affected by different post-translational modifications such that a given radiotracer may be able to bind NFTs but not to other ultrastructural tau conformations, such as straight (SF) or randomly coiled filaments (RCFs). Additionally, tau expression as 3R and 4R isoforms can strongly modulate the availability of binding pockets, since these different isoforms can assume distinct structural conformations.

In this context, the combination of tau tracers and genetic manipulation to achieve transgenic rodent models expressing human tau with a given conformation/isoform may accelerate our understanding of the mechanisms subserving tau-mediated neurodegeneration and clinical progression across the spectrum of tauopathies.

### Tracking tau pathology in small animals

Since only human tau protein assumes the structural conformation rendering NFTs, transgenic models expressing human tau are *sine*-*qua*-*non* for testing new imaging agents with high translation value for human diseases [9]. Further, a high degree of homology and functional similarity is observed between humans and rodents in brain areas recognized as predilection sites for tau pathology, including the hippocampus [36-38].

Tau transgenic models, combined with behavioral assessment and *in vivo* imaging using microPET stands as a unique strategy for establishing the clinico-pathological correlations needed for testing the efficacy of novel disease-modifying drugs. Additionally, *in vivo* tracking of tau pathology in transgenic rodent models will provide new insights with high translational value owing to the spatiotemporal pattern of propagation similar to that observed in human tauopathies [37] (Figure 2b). In addition to occupying an important role in the full validation of tau-imaging tracers, microPET has the potential to provide high-resolution dynamic images that will estimate the content of tau aggregates via pharmacokinetic analysis. Finally, microPET can deliver crucial information regarding tracer kinetics, requisite for both the development of novel tau radiopharmaceuticals and in the context of imaging analysis.

### Clinical utility

It is expected that tau-imaging agents will contribute to the early diagnosis of tauopathies. In addition, these imaging agents will significantly expand our knowledge regarding the propagation of tau protein in the human brain as well as its relationships with clinical features of the various tauopathies. Given the numerous drugs aiming to halt the progression of tau pathology, it is also expected that these ligands will allow for the monitoring of treatment effects of novel anti-tau therapies (see review Giacobini et al. [39]). Yet, there remains a significant amount of research to be conducted in order to establish the advantages and limitations of tau-imaging agents. Indeed, well-controlled microPET studies in animal models are necessary for the validation and development of tau specific PET agents with less non-specific binding and favorable kinetics (Table 1).

**Table 1:** Tau imaging agents: from basic to clinical applications

**Basic research**	The combination of Tau imaging agents and transgenic models can allow for:
	a) Non-invasive longitudinal tracking of tau pathology
	b) Longitudinal assessment of behavior as a function of tau pathology
	c) Determination of pharmacokinetic properties
	d) Development of novel radiopharmaceuticals
	e) Development of theoretical models regarding progression of tau pathology
**Clinical applications**	The use of Tau imaging agents in the clinical approach can allow for:
	a) Early diagnosis
	b) Differential diagnosis
	c) Follow-up of cognitive decline as function of tau pathology progression
	d) Monitoring of treatment effectiveness of novel anti-tau and associated therapies
	e) Development of theoretical models regarding tau pathology progression
	f) Estimation of sample size and endpoints in the context of clinical trials

### Concluding remarks

PET in tau transgenic models allows for longitudinal assessment and thus is ideally suited for following tau pathology progression and/or response to disease-modifying drugs. Simultaneously, the use of the same animal for PET imaging and behavioral assessments eliminates the between-subject variability and will provide key insights on the role of tau in neurodegeneration and associated cognitive decline. Finally, these results can rapidly be translated to human research, delivering novel therapeutic strategies, mechanistic insights, predictive models and biomarkers.

## Competing interest

The authors declare that they have no competing interest.

## Authors’ contribution

ERZ and AL drafted the manuscript and designed the figures. VB critically revised the manuscript. SG and PRN guided and supervised the work. All authors read and approved the final manuscript.
